# Evaluating the role of type 2 diabetes mellitus in rotator cuff tendinopathy: Development and analysis of a novel rat model

**DOI:** 10.3389/fendo.2022.1042878

**Published:** 2022-10-10

**Authors:** Kuishuai Xu, Liang Zhang, Zhongkai Ren, Tianrui Wang, Yingze Zhang, Xia Zhao, Tengbo Yu

**Affiliations:** ^1^ Department of Sports Medicine, the Affiliated Hospital of Qingdao University, Qingdao, China; ^2^ Department of Abdominal Ultrasound, the Affiliated Hospital of Qingdao University, Qingdao, China; ^3^ Department of Traumatology, the Affiliated Hospital of Qingdao University, Qingdao, China

**Keywords:** rotator cuff, diabetes, tendinopathy, mechanics, animal model

## Abstract

**Objective:**

To establish and validate an intact rotator cuff rat model for exploring the pathophysiological effects of type 2 diabetes on the rotator cuff tendon *in vivo*.

**Methods:**

A total of 45 adult male rats were randomly divided into a control group (n = 9) and type 2 diabetes group (n=36). The rats were sacrificed at 2 weeks (T2DM-2w group, n=9), 4 weeks (T2DM-4w group, n=9), 8 weeks (T2DM-8w group, n=9), and 12 weeks (T2DM-12w group, n=9) after successful modeling of type 2 diabetes. Bilateral shoulder samples were collected for gross observation and measurement, protein expression(enzyme-linked immunosorbent assay,ELISA), histological evaluation, biomechanical testing, and gene expression (real-time quantitative polymerase chain reaction, qRT-PCR).

**Results:**

Protein expression showed that the expression of IL-6 and Advanced glycation end products (AGEs)in serum increased in type 2 diabetic group compared with the non-diabetic group. Histologically, collagen fibers in rotator cuff tendons of type 2 diabetic rats were disorganized, ruptured, and with scar hyperplasia, neovascularization, and extracellular matrix disturbances, while Bonar score showed significant and continuously aggravated tendinopathy over 12 weeks. The biomechanical evaluation showed that the ultimate load of rotator cuff tendons in type 2 diabetic rats gradually decreased, and the ultimate load was negatively correlated with AGEs content. Gene expression analysis showed increased expression of genes associated with matrix remodeling (COL-1A1), tendon development (TNC), and fatty infiltration (FABP4) in tendon specimens from the type 2 diabetic group.

**Conclusion:**

Persistent type 2 diabetes is associated with the rupture of collagen fiber structure, disturbance in the extracellular matrix, and biomechanical decline of the rotator cuff tendon. The establishment of this new rat model of rotator cuff tendinopathy provides a valuable research basis for studying the cellular and molecular mechanisms of diabetes-induced rotator cuff tendinopathy.

## Introduction

Tendinopathy is a common muscle tissue disease with multifactorial pathogenesis that has not yet been fully elucidated. It usually occurs due to overuse, metabolic disturbances, and impact of other factors related to tendon microinjury, one of which is diabetes as a metabolic disorder. Tendinopathy is a common musculoskeletal complication of diabetes mellitus (DM) (1). The effects of type 2 diabetes on tendon structure and homeostasis are often overlooked before serious complications or acute injuries occur because the effects of type 2 diabetes on tendons tend to persist, and tendon-related diseases are often difficult to detect and treat in advance before acute tears occur. The lack of continuous effects of type 2 diabetes before acute injuries leads to pathological changes in tendon tissue, especially on the rotator cuff tendon.

DM is one of the risk factors for rotator cuff injury, and patients with type 2 DM are at a higher risk of tendon rupture (2, 3). The prognosis and healing rates of the rotator cuff after repair are poor (4). Diabetic rats have increased rotator cuff fatty infiltration, poor biomechanics (5), and accelerated steatosis after rotator cuff injury (4). In addition, the detrimental effects of diabetes persist even after the repair of rotator cuff injury, and persistent hyperglycemia impairs tendon-to-bone healing after rotator cuff repair in a rat model, resulting in worse biomechanics and histology (6), which may be associated with the let-7b-5p/CFTR pathway (7). However, whether this leads to poor biomechanics is still controversial. Another study concluded that hyperglycemia alone does not affect the biomechanical properties of the rotator cuff but induces a chronic inflammatory response (5). An increasing number of clinical studies have shown that the incidence of tendinopathy is significantly increased in patients with type 2 diabetes compared with non-diabetic patients (8, 9), and tendinopathy is associated with the duration of type 2 diabetes (10–12). Unfortunately, previous findings have been dominated by clinical studies, none of which described in detail how type 2 diabetes impacts tendon structure, extracellular matrix homeostasis, and biomechanics at different stages of the course of the disease, particularly the supraspinatus tendon of the rotator cuff. In addition, previous studies lacked comprehensive and systematic evaluation and description of time window tendinopathy manifests after diabetes onset.

To this end, we investigated an animal model with an intact rotator cuff. The aim of the present study was to evaluate the effects of type 2 diabetes on rotator cuff tendon structure and homeostasis over the course of the disease by ELISA, histology, biomechanics, and qTR-PCR in a rat model at various time points after induction of non-diabetic and type 2 diabetes, thus providing a more appropriate animal model and theoretical basis for exploring the pathophysiologica l effects of type 2 diabetes on the rotator cuff tendon.

## Methods

### Experimental animals

A total of 45 adult male clean Sprague-Dawley rats (6 weeks old, 200-250 g, Beijing Vital River) were used for animal modeling. All the animals were housed in an environment with a temperature of 23 ± 2 °C, relative humidity of 50 ± 5%, and a light/dark cycle of 12/12 hr and had free access to food and water. All animal studies (including the mice euthanasia procedure) were done in compliance with the regulations and guidelines of Qingdao University institutional animal care and conducted according to the AAALAC and the IACUC guidelines.

### Animal grouping

**Figure 1:**
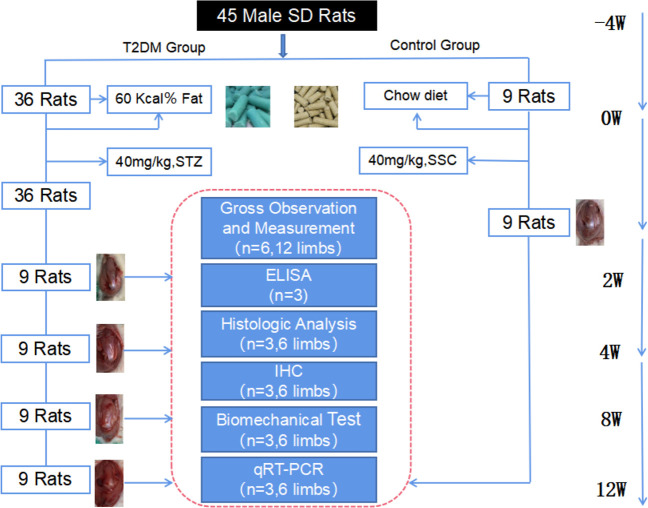
Flow chart of the experimental design.

A total of 45 rats were numbered and randomly divided into non-diabetic group (NDM group, n = 9) and type 2 diabetic group (T2DM group, n = 36) by random number table. According to different sampling time, rats in type 2 diabetic group were divided into 2-week diabetic group (T2DM-2w group, n = 9), 4-week diabetic group (T2DM-4w group, n = 9), 8-week diabetic group (T2DM-8w group, n = 9) and 12-week diabetic group (T2DM-12w group, n = 9),NDM group rats sacrifice times were the same as T2DM-12w group rats. After purchase, the rats were fed a normal diet in the animal laboratory of Qingdao University, and the diabetic group was fed a high-fat diet for 1 month. The rats in both groups had free access to water, and the bedding was replaced daily. The experimental animal grouping and study design are shown in **Figure 1**.

### Constructing a rat model of type 2 diabetes

In order to ensure normoglycemia before modeling, blood glucose was measured before the induction of type 2 diabetes in both groups. After 4 weeks of high-fat diet intervention, rats in the type 2 diabetes group were fasted for 12 h, which was followed by intraperitoneal injection of Streptozotocinn(STZ) solution (injection dose of 40 mg/kg) and continuation of high-fat feeding (13). The non-diabetic group was injected with the same amount of citric acid buffer. Three days later, blood samples were collected at fixed times each day, and rats were considered a successful diabetic rat model when fasting blood glucose levels were measured three consecutive times ≥16.7 mmol/L for three consecutive days (14). This model was eliminated and supplemented in a timely manner when random blood glucose was < 16.7 mmol/L.

### Obtaining supraspinatus tendon-humerus specimens

At the 2^nd^, 4^th^, 8^th,^ and 12^th^ week after modeling, the left forelimb of diabetic rats was divided from the elbow joint and scapula, the excess muscle tissue was removed, the complete supraspinatus tendon-humerus structure was obtained, and the right specimen was removed by following the same method. Tissue samples were stored in a -80° freezer after collection.

### Gross tissue assessment and measurement of the supraspinatus tendon

The quality and color of the supraspinatus tendon in the rotator cuff and its adhesion to surrounding tissues were observed. Supraspinatus tendon width, thickness, and weight were measured using a digital micrometer (Syntek, Zhejiang, China).

### ELISA

A total of 1.5 mL of venous blood was drawn from the orbital venous plexus of fasting rats in the morning at 2, 4, 8, and 12 weeks after the establishment of the model in the non-diabetic group and type 2 DM. It was rapidly transferred to a biochemical tube and centrifuged at 2500 r/min for 20 min. The supernatant was transferred and stored in a -80°C freezer for examination. Serum levels of IL-6, PGE2, and AGEs were measured by referring to the ELISA kit instructions for rat IL-6 (EK306/3-48, MultiSciences, Zhejiang, China), PGE2 (EK8103/2-48, MultiSciences, Zhejiang, China), and AGEs (CSB-E09413r, Cusabio, Wuhan, China).

### Histological evaluation

Bilateral shoulder joints were taken from rats in each group. The muscle parts of the humerus and supraspinatus muscles of the samples were immediately removed, after which the supraspinatus tendons were fixed in paraformaldehyde for 24 h. The tissues were dehydrated and embedded in paraffin with an automatic tissue processing device. The embedded samples were sectioned at a thickness of 5 mm parallel to the direction of the tendon course. Three stains were performed: hematoxylin and eosin (HampE), picrosirius red (S8060, Solarbio, Beijing, China), and alcian blue staining (DG0041, Leagene, Beijing, China), and the relevant procedures were performed according to previous literature (15).

For immunohistochemical staining, 4 µm sections were dewaxed at 65°C, dewaxed in xylene, treated with graded ethanol, blocked with 3% hydrogen peroxide for 10 min, infiltrated for 15 min, incubated in goat serum, and immediately reacted with primary antibody at 4°C for ≥ 16 h (overnight). Then, samples were washed three times with PBS solution, incubated with the corresponding secondary antibody conjugated to horseradish peroxidase for 30 min at room temperature, and then washed three times with PBS solution. DAB chromogenic solution (volume ratio of concentrated DAB chromogenic solution to buffer 1:500) was reacted for 1 to 5 minutes and washed with pure water 3 to 4 times; Specimens were stained with hematoxylin-eosin and sequentially placed in ethanol at concentrations of 80%, 90%, and 100% for dehydration.Finally, xylene transparent, neutral gum mounting was performedxylene transparency, and neutral gum mounting. Used antibodies included biglycan (Biglycan) antibody (K008381P, Solarbio, Beijing), factor VIII antibody (AB275376, Abcam, UK), MMP-3 (K111452P, Solarbio, Beijing), and IL-6 (KOO9639P, Solarbio, Beijing). HampE, immunohistochemistry, and Alcian blue staining were observed using a Nikon E100 microscope (E100, Nikon, Japan), tissue imaging with Sirius red staining was observed using a polarized light microscope (DM4500, Leica, Germany), and scanning of images was performed using a panoramic scanner (3DHISTECH P250 FLASH, Beijing, China). After staining, five sections were selected from each sample, and three fields of view were selected for densitometric value (OD) analysis at ×400 fields in the tendon region for each section. Microscopically, positive brown positive reaction areas were selected, and positive expression was semiquantitatively analyzed by ImagePro Plus 8.0 software in order to detect cumulative optical density (IOD) and positive area, after which the ratio of the two was taken as the expression level.

Tendinopathy was graded using a modified semi-quantitative Bonar scoring system as previously described (16). A histology scoring system was used for semi-quantitative assessment of the repair interface. The total histological score was calculated by two investigators (KSX and LZ), with higher histological scores indicating more severe tendinopathy.

### Biomechanical analysis

Biomechanical tests were performed on the bilateral shoulders of rats in each group (3 rats per group), and the humeral muscles and tissues around the humeral head were carefully removed. Only specimens of the supraspinatus and humeral complex were preserved, and the musculature on the supraspinatus tendon was curetted with a scalpel. The humerus was firmly fixed on the base of the testing machine using polymethyl methacrylate (Thermo Fisher Scientific ‘s Products, Shanghai, China) (17), and the tendons were flattened, fixed using sandpaper and cyanoacrylate glue (Dongxin’ s Products, Shenzhen, China), and then placed in customized serrated grips. After preconditioning with 0.1 N, the traction load was gradually increased until the tendon was completely ruptured at a fixation speed of 10 mm/min (18, 19). Ultimate load and stiffness were recorded. In the load-displacement curve, the slope of its linear segment was used to express its stiffness value.

### qRT-PCR analysis

After sampling at predetermined time points, bilateral supraspinatus tendons and muscles were carefully separated, quickly snap frozen in liquid nitrogen, and then stored in a -80°C freezer for testing. Total RNA was extracted by the Trizol method, RNA quality was measured by spectrophotometer, and mRNA expression levels were measured by RT-qPCR. The corresponding cDNA was obtained by reverse transcription of the resulting RNA samples, and the qRT-PCR system was prepared according to the instructions of the SYBR Green kit (Q711-02, Vazyme, Nanjing, China) in this experiment. The cDNA, upstream and downstream primers, SYBR GREEN master mix, and ddH2O were reacted, and quantitative fluorescence analysis was performed using a real-time fluorescence quantifier (Thermo Fisher Scientific, Shanghai, China). Collagen1A1 (COL1A1), Tenascin C (TNC), RUNX2, Scleraxis (SCX), SOX9, and Tenmodulin (TNMD) were tested in supraspinatus tendon samples. Fatty acid binding protein 4 (FABP4) and peroxisome proliferator-activated receptor gamma (PPARγ) were measured in muscle specimens. Three independent replicates were performed for each sample gene, and Gapdh was used as an internal reference value to calculate and count the target gene using the 2^-ΔΔCT^ method. The experimental results were averaged. Primer sequences are shown in **Table 1**.

**Table 1:** Primer information of RT-PCR gene.

Gene	Sequence(5’-3’)	Product Length/bp
GAPDH	Gapdh-F: CTGCCTTCTCTTGTGACAAAGTG	148
	Gapdh-R: TTGATGACCAGCTTCCCATTCTC	
COL1a1	Col1a1-F: ATCAAGGTCTACTGCAACATGGA	119
	Col1a1-R: AACCAGACATGCTTCTTCTCCTT	
TNMD	TNMD-F: AACAGTCAGTGATTTGGGTTCCT	104
	TNMD-R: CCAGTACATAGTCACATTGTCGC	
TNC	TNC-F: TATGACAAGGACACAGACTCAGC	112
	TNC-R: TATTGTCCCCATATCTGCCCATC	
SOX9	SOX9-F:GATAAATTCCCAGTGTGCATCCG	120
	SOX9-R: CTTGACGTGTGGCTTGTTCTTG	
RUNX2	RUNX2-F:ATGGCCGGGAATGATGAGAACTA	101
	RUNX2-R:CGGCCTACAAATCTCAGATCGTT	
FABP4	FABP4-F:CCCAACTTGATCATCAGCGTAGA	113
	FABP4-R:GGGGTGATTTCATCGAATTCCAC	
SCX	SCX-F:CGAAAAACCCTGTCGTGTTCATG	179
	SCX-R:CCGTGTTCACGCTGTTGGTG	
PPARγ	PPAR-F:CTCCAGAAGATGACAGACCTCAG	117
	PPARγ-R:CTTGTAGATCTCCTGGAGCAGAG	

COL1A1, collagen1A1; TNMD, tenomodulin; SCX, scleraxis; TNC, Tenascin C; RUNX2, runt-related transcription factor 2; FABP4, fatty acid binding protein 4; PPARγ,peroxisome proliferator-activated receptor gamma.

### Statistical analysis

All data analyses were performed using SPSS 21.0 (IBM, Armonk, NY) and GraphPad Prism 8.0 (La Jolla, CA). Student’s t-test was used to perform statistical comparisons between biomechanical, ELISA, Banor score, and qRT-PCR data at each time point, and continuous data were presented as mean ± standard deviation. Figures were plotted using Prism 8.0 with mean ± standard error of the mean (SEM). A P value < 0.05 indicated statistical significance.

## Results

### Evaluation of a rat model of type 2 diabetes

**Figure 2:**
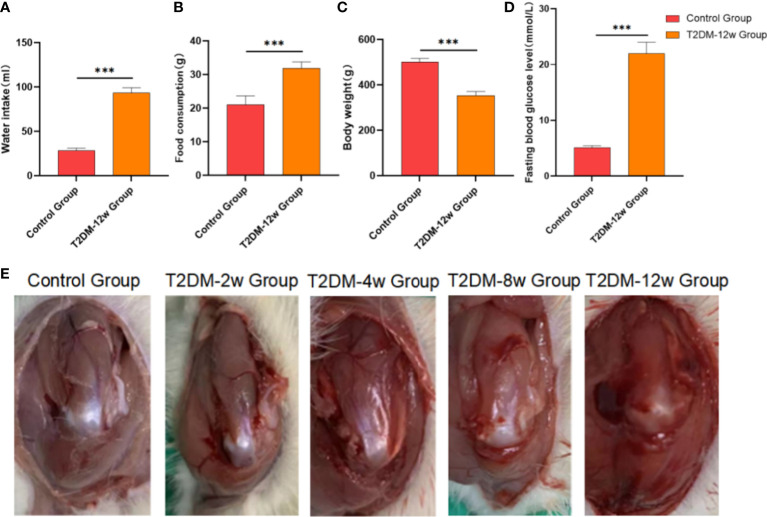
Evaluation of a rat model type 2 diabetes. **(A)** Water intake. **(B)** Food consumption. **(C)** Body weight. **(D)** Fasting bloiod glucose level. **(E)** Gross observation of supraspinatus tendon of rotator cuff in each group. ***p < 0.001 versus Control group. The values are presented as means, with the error bars depicting the standard deviation.

Rats in the T2DM-12w group with random blood glucose > 16.7 mmol/L after modeling showed significant symptoms of polydipsia and polyphagia. They also gained weight slowly compared with the control group. The detailed results are shown in **Figures 2A–D**.

### Gross tissue observation and measurement of supraspinatus tendon in type 2 diabetic rats

None of the rats in any of the groups died. Compared with the control group, the supraspinatus tendon color became darker, and the gloss gradually worsened at 2 weeks, 4 weeks, 8 weeks, and 12 weeks after modeling in diabetic rats. Detailed results are shown in **Figure 2E**.

### Type 2 DM causes persistent histologic tendinopathy of the rotator cuff

**Figure 3:**
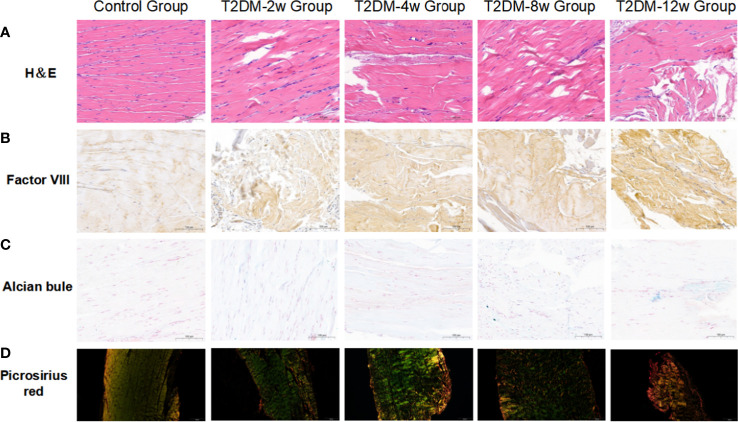
Representative images of histology. **(A)** Hematoxylin and eosin(H&E) staining(40X). **(B)** Immunohistochemical staining of factor VIII(40X). **(C)** Alcian-blue staining(40X). **(D)** Picrosirius-red staining(10X). Scale bars depict 100µm.

**Figure 4:**
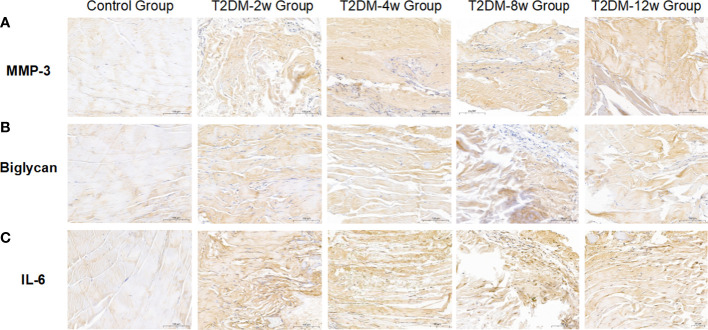
Representative images of immunohistochemical staining. **(A)** MMP-3 (40X). **(B)** Biglycan (40X). **(C)** II-6 (40X). Scale bars depict 100µm.

**Figure 5:**
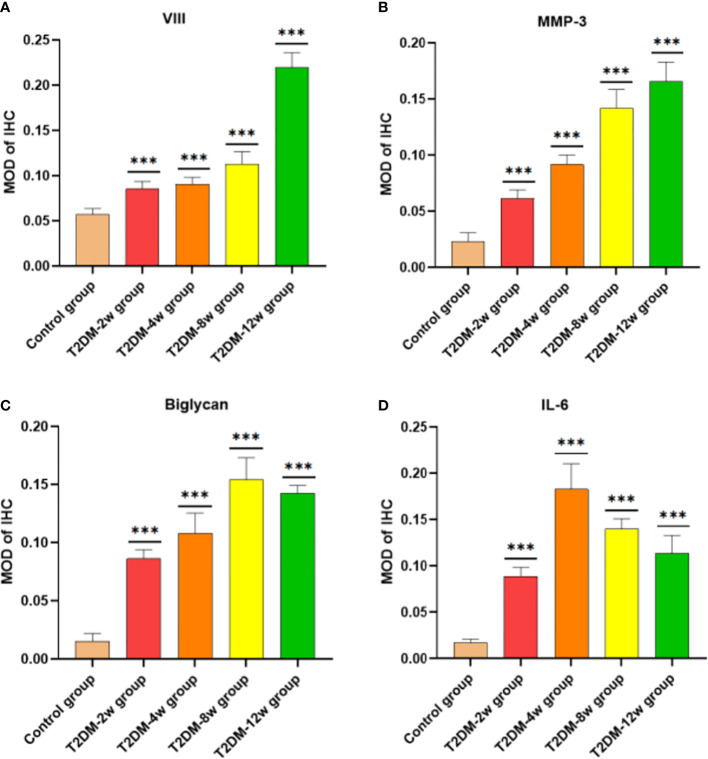
Comparison of semi-quantitative MOD after immunohistochemical staining. **(A)** VII. **(B)** MMP-3 (40X). **(C)** Biglycan (40X). **(D)** il-6 (40X). MOD is the ratio of cumulative optical density value (IOD) and positive area (AREA). ***p < 0.001 versus Control group. The values are presented as means, with the error bars depicting the standard deviation.

**Figure 6:**
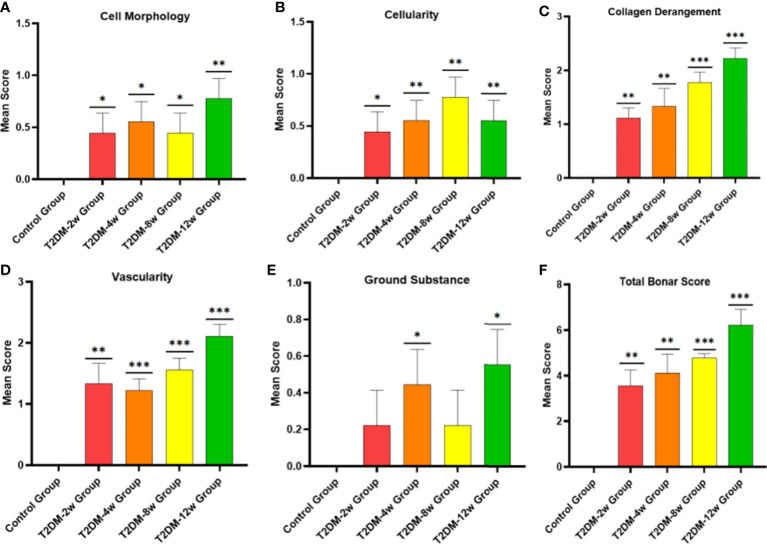
Histological specimens were scored using a modified Bonar scoring scheme. **(A-F)**, semiquantitative histological scores showed persistent tendinopathy changes in the rotator cuff tendon due to type 2 diabetes. *p < 0.05 versus Control group. **p < 0.01 versus Control group. ***p 0.001 versus Control group. The values are presented as means, with the error bars depicting the standard deviation.

Histological findings revealed that sparse arrangement and microtears of collagen fibers were first detected 2 weeks after diabetes induction. As the disease progressed, the number of supraspinatus tendon cells increased, volume of matrix increased, chondroid tissue formation was observed, and tendon collagen fibers showed significant tears and inflammatory cell infiltration by 12 weeks (**Figures 3A, C, D**). Supraspinatus tendon showed no significant fatty infiltration and calcification. Immunohistochemical results further showed that protein expression involving tissue repair (type I collagen), scar formation (Biglycan), angiogenesis (VIII), and extracellular matrix remodeling marker (MMP-3) was increased in the tendon tissue of rats with type 2 diabetes (**Figures 3B**, **4A–C**), and optical density values (OD) were significantly increased compared with the non-diabetic group (**Figures 5A–D**). Increased tissue repair, scarring, newly formed vascular invasion, and extracellular matrix remodeling suggested that diabetes induced sustained changes in the rotator cuff tendon. As it was challenging to find manifestations of rotator cuff tendon degeneration in non-diabetic rats, we determined the modified Bonar score as 0 in non-diabetic rats. Type 2 diabetes can lead to persistent tendinopathy for at least 12 weeks after induction. The mean modified Bonar score was significantly higher in the type 2 diabetes group than in the control group (T2DM-2w group: 3.56 ± 0.69; T2DM-4w group: 4.11 ± 0.84; T2DM-8w group: 4.78 ± 0.19. T2DM-12w group: 6.22 ± 0.69). Detailed results are shown in **Figure 6**.

### Type 2 diabetes leads to an increase in serum inflammatory factors and a decrease in the ultimate load of the supraspinatus tendon of the rotator cuff

**Figure 7:**
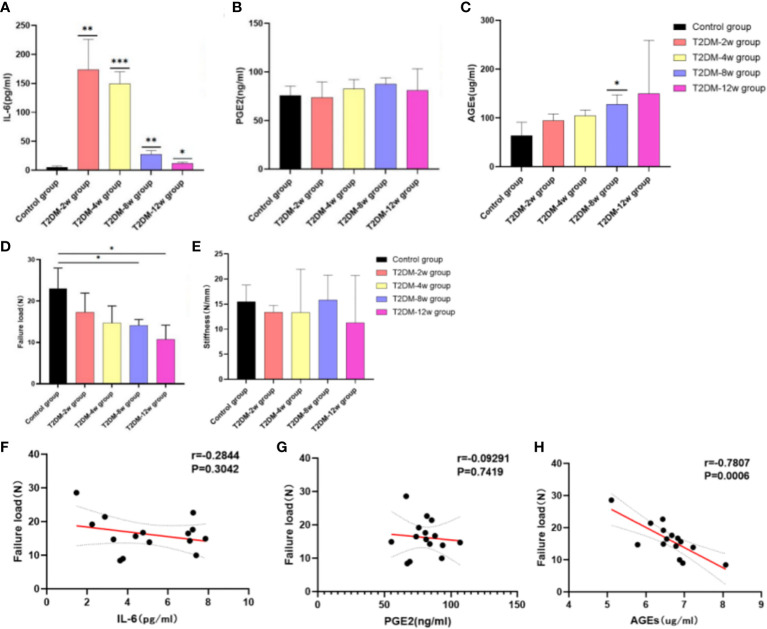
Results of ELISA and biomechanical testing. **(A)** Expression of IL-6. **(B)** PGE2 expression. **(C)** Expression of AGEs. **(D)** Failure load. **(E)** Stiffness. **(F)** Correlation analysis between IL-6 and Failure load. **(G)** Correlation analysis between PGE2 and Failure load. **(H)** Correlation analysis between AGEs and Failure load. Pearson correlation analysis was used to analyze the correlation between IL-6, PGE2, AGEs and Failure Load. *p < 0.05 versus Control group, **0.01 versus Control group. ***p < 0.001 versus Control group. The values are presented as means, with the error bars depicting the standard deviation.

ELISA results showed that the expression of serum IL-6 was highest at 2 weeks after induction of type 2 diabetes, followed by a gradual decrease in expression compared with the non-diabetic group (**Figure 7A** **)** (p < 0.05). There was also a clear upward trend in PGE2 expression, but there was no statistically significant difference (**Figure 7B**) (p > 0.05). The expression of serum AGEs showed a significant increasing trend within 12 weeks after induction of type 2 diabetes; the observed difference was statistically significant (**Figure 7C**) (p < 0.05).

In all specimens, the site of destruction of the supraspinatus tendon was located in the middle of the tendon. The biomechanical evaluation showed that the ultimate load (**Figure 7D**) and stiffness (**Figure 7E**) of the supraspinatus tendon had a significant decreasing trend in type 2 diabetic rats compared with the non-diabetic group, and tissue stiffness was restored at 8 weeks. Ultimate load and stiffness were not significantly different between diabetic and non-diabetic groups within 4 weeks after induction of type 2 diabetes (P < 0.05). At 8 (14.11 ± 1.43 N) and 12 weeks (10.7 ± 3.47 N) after induction of type 2 diabetes, the mean ultimate load breaking in the non-diabetic group (23.06 ± 4.92 N) was significantly higher than that in the diabetic group (p < 0.05). However, there was no significant difference in stiffness of the supraspinatus tendon between the two groups (p > 0.05) In addition, there was a significant negative correlation between the ultimate load of the supraspinatus tendon and the expression of serum AGEs (r =-0.7807; p = 0.0006) (**Figure 7H**), but no correlation with IL-6 (r =-0.2844; p = 0.3042) and PGE2 (r =-0.09291; p = 0.7419) (**Figures 7F, G**).

### Type 2 diabetes causes persistent changes in gene expression profiles of affected tendons

**Figure 8:**
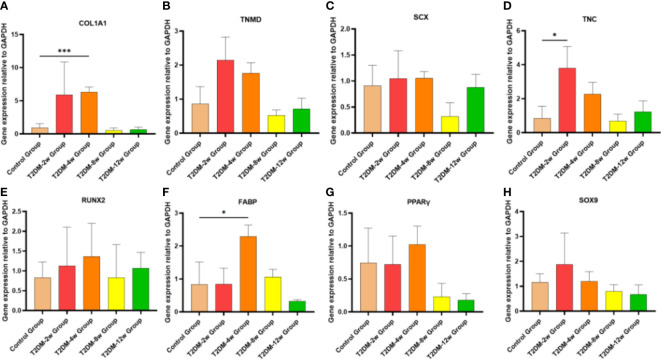
Type 2 diabetes leads to persistent alterations in gene expression in tendon tissue. **(A–H)**. qRT-PCR analysis of suspraspinatus tendon associated gene expression (COl1A1, TNMD, SCX, TNC, RUNX2, FABP, PPARγ and SOX9) in diabetic and nondiabetic subjects. COL1A1, collagen1A1;TNMD, tenomodulin; SCX,scleraxis;TNC:Tenascin C; RUNX2, runt-related transcription factor 2; FABP4, fatty acid binding protein 4; PPARγ, peroxisome proliferator-activated receptor gamma. Error bars depict standard error. *p < 0.05 versus Control group. ***p < 0.001 versus Control group. The values are presented as means, with the error bars depicting the standard deviation.

Gene expression trends showed high variability within the diabetic group; however, these qRT-PCR results could still provide preliminary observations on gene expression in type 2 diabetes leading to tendinopathy (**Figures 8A–H**). Compared with the non-diabetic group, the expression levels of genes involved in tissue repair (COL1A), tenogenesis (TNC, TNMD, SCX), osteogenesis (RUNX2), and chondrogenesis (SOX9) of the supraspinatus tendon of the rotator cuff showed an increasing trend after induction of type 2 diabetes, where the TNC mRNA expression levels of the supraspinatus tendon were significantly increased in the 2-week group after induction of type 2 diabetes (p < 0.05), and the COL1A1 mRNA expression levels of the supraspinatus tendon were significantly increased in the 4-week group after induction of type 2 diabetes (p < 0.001). Fat infiltration-related genes (FABP and PPARγ) expression tended to increase in supraspinatus muscle specimens, and fat infiltration developed gradually over time, with FABP4 mRNA expression levels significantly increasing in the 2-week group after type 2 diabetes induction (p < 0.05). Although some genes expression (TNMD, SCX, TNMD, SCX, SOX9, PPARγ) in the diabetic group were not statistically significant compared with the non-diabetic group (p > 0.05), the gradual increase in tendon gene expression from 2 weeks to 12 weeks after induction of type 2 diabetes showed that the hyperglycemic microenvironment produced long-term chronic changes in the supraspinatus muscle of rats.

## Discussion

In the current study, two important findings emerged using protein expression, histological assessment, biomechanical testing, and gene expression. First, we developed a rat model of rotator cuff tendinopathy induced by type 2 diabetes in rats. To the best of our knowledge, this is the first systematic study that evaluated the role of type 2 diabetes in rotator cuff tendinopathy. In addition, we divided and sampled experimental animals according to the duration of disease after induction of type 2 diabetes and confirmed that type 2 diabetes persistently leads to collagen fiber tears in the rotator cuff tendon, increased histological scores, extracellular matrix disturbances, and biomechanical decline. Moreover, we believe this is the first basic study that assessed the impact of type 2 diabetes on rotator cuff tendinopathy with the progression of the disease.

Two weeks after induction in type 2 diabetic rats, we were the first to detect a sparse arrangement of collagen fibers and a small number of microtears, which are the first features of rotator cuff tendinopathy. It is well-known that tendon structures in diabetic patients show disorganized collagen fibers and microtears (20), tendon thickening (21), and calcification (22) compared with normal tendons. As the disease progresses, collagen fibers become more disorganized and tear further increase. Previous studies reported histopathological changes associated with diabetic tendinopathy. In an earlier study, Kent et al. found that a high glucose environment alters collagen alignment in isolated tendons (23). Ahmed et al. reported similar results, with a marked reduction in collagen organization in injured tendons in diabetic rats (24). Even long-term insulin resistance adversely affects tendons, resulting in decreased collagen fiber density (25, 26), collagen fiber disorganization, and increased vascularity (27). Using immunohistochemical staining, similar studies have confirmed our experimental results that vascular endothelial growth factor expression is increased in tendons of diabetic rats, which may lead to altered vascularization (20). Our results are similar to previous studies in that we found more neovascularization within collagen fibers in the supraspinatus tendon of the rotator cuff as the course of the disease progressed.

In the current study, gene expression analysis confirmed that COL1A1 and TNC in the supraspinatus tendon of the rotator cuff in type 2 diabetic rats continued to increase in the first four weeks, after which they showed a trend of continuously decreased expression, indicating that there is a continuous remodeling process in the supraspinatus tendon in response to persistent microdamage caused by hyperglycemic conditions. The main component of tendon ECM is type I collagen, which is sensitive to the process of glucose oxidation, while the accumulation of glycogen during glucose oxidation further alters the quality of tendon ECM (28, 29). In addition, severe glycation of type I collagen and other matrix proteins occurs in the tendons of diabetic patients (30), further leading to reduced ECM remodeling (31). In a similar study, Lin et al. (32) found that expression of collagen I (Col1) and tenocyte markers were reduced in cultured tenocytes under high glucose for 48 hours.

Aberrant matrix remodeling is an important part of the pathology leading to the development and progression of tendinopathy. Immunohistochemical results showed that increased MMP-3 expression means dysregulated extracellular matrix turnover and remodeling and increased collagen degradation, both of which are direct evidence of extracellular matrix degradation and disturbance. In their study, Ueda et al. (33) and Tsai et al. (34) found that culturing rat Achilles tendons in high glucose for 72 hours caused increased expression of matrix metalloproteinases and the pro-inflammatory cytokine interleukin-6. In our study, diabetes increased the body’s inflammatory response with the progression of the disease. We also detected higher expression of AGEs and IL-6 in the serumof diabetic rats than non-diabetic rats. In addition, immunohistochemical results revealed that the expression of IL-6 levels in the supraspinatus tendon of the rotator cuff was increased in type 2 diabetic rats, and the expression of inflammatory factors, as the first manifestation of tendinopathy, may be related to the disturbance of the extracellular matrix. We also found that AGEs expression in serum was significantly higher in type 2 diabetic rats than in non-diabetic rats; however, we did not investigate AGEs expression in tendons. Previous studies revealed that accumulation of AGEs in collagen could lead to degradation of ECM (35) and matrix metalloproteinases (MMPs) expression levels (11), while expression of MMPs further leads to degradation of type 1 collagen (36), which is consistent with our findings. Collectively, these studies suggest that a sustained hyperglycemic state may impair the homeostasis of tenocytes and extracellular matrix, thus further leading to high expression of pro-inflammatory and profibrotic mediators.

Biomechanical tests showed that the rotator cuff tendons of type 2 diabetic rats tended to decrease as the disease progressed, and the difference in ultimate load was statistically significant by week 8, which was the earliest time point at which biomechanical differences were observed. Previous studies have reported conflicting conclusions regarding the effect of diabetes on tendon mechanics. The intact rotator cuff in diabetic rats was biomechanically worse compared with normal rats (6). Another study reported the opposite conclusion, i.e., that hyperglycemia alone does not reduce the mechanical properties of the shoulder (5); nevertheless, our findings were more in line with the former study, and collagen fiber tear and disturbance of the extracellular matrix may be important causes of biomechanical deterioration. Previous studies have investigated and interpreted the decline in biomechanics, with a linear increase in intermolecular distance in glycated tendons (28), reporting that abnormal cross-linking of collagen fibers may occupy space in the ECM, thus affecting biomechanical properties (37). In addition, the high-glucose microenvironment leads to abnormal cross-linking and disarrangement of collagen fibers, resulting in biomechanical decline (38), and these structural abnormalities may be attributed to the deposition of AGEs (39). This conclusion was further confirmed by our correlation analysis between AGEs and ultimate load, where the deposition of AGEs may be one of the factors leading to the biomechanical decline of tendon tissue. In addition, we investigated the repair of scar tissue after supraspinatus tendon injury in type 2 diabetic rats. By immunohistochemical staining, Biglycan protein expression was significantly increased in the rotator cuff tendon of diabetic rats, indicating that the scar tissue reaction was enhanced in the supraspinatus tendon of type 2 diabetic rats. This would affect the reconstruction of tendon mechanical structure and performance recovery and could be related to the increased risk of tendon rupture and decreased biomechanical properties.

The current study has several limitations. First, the establishment of a rat model of type 2 diabetes cannot completely simulate the pathological state of the human rotator cuff tendon. Nonetheless, this model mimics the natural process of rotator cuff tendinopathy caused by type 2 diabetes to a certain extent, In particular, the tissue microenvironment continues to change at different stages, thus having great value for further study of the causes of rotator cuff tendon injury and the molecular mechanism of healing. Second, we investigated changes in rotator cuff tendon structure and homeostasis throughout the disease, while the molecular mechanisms involved were not explored. Finally, there was variability between samples and variation between individuals, and future studies with bigger sample sizes are needed.

## Conclusion

As type 2 diabetes progressed, the development of rotator cuff tendinopathy was demonstrated in a rat model. Moreover, we found persistent histological, biomechanical, and gene expression changes. This model provides a valuable research basis for further evaluation of the underlying cellular and molecular mechanisms of rotator cuff tendinopathy caused by type 2 diabetes.

## Data availability statement

The original contributions presented in the study are included in the article. Further inquiries can be directed to the corresponding authors.

## Ethics statement

The animal study was reviewed and approved by Ethics Committee for Laboratory Animal Welfare of Qingdao University.

## Author contributions

Authors KSX, XZ, and TBY designed the study; LZ and ZKR analyzed the data; KSX and TRW wrote the manuscript; YZZ and TBY supervised the study. All authors approved the final manuscript as submitted and agree to be accountable for all aspects of the work. All authors read and approved the final manuscript.

## Acknowledgments

We would like to thank Zhengyi Shan, Department of Pathology, the Affiliated Hospital of Qingdao University, for his important contribution to specimen preparation and histological analysis required to complete this project.

## Conflict of interest

The authors declare that the research was conducted in the absence of any commercial or financial relationships that could be construed as a potential conflict of interest.

## Publisher’s note

All claims expressed in this article are solely those of the authors and do not necessarily represent those of their affiliated organizations, or those of the publisher, the editors and the reviewers. Any product that may be evaluated in this article, or claim that may be made by its manufacturer, is not guaranteed or endorsed by the publisher.
